# A Countrywide Survey of *hrp2/3* Deletions and *kelch13* Mutations Co-occurrence in Ethiopia

**DOI:** 10.1093/infdis/jiae373

**Published:** 2024-07-31

**Authors:** Claire Kamaliddin, Jack Burke-Gaffney, Shoaib Ashraf, Daniel Castañeda-Mogollón, Aderaw Adamu, Bacha Mekonen Tefa, Ayesha Wijesinghe, Enaara Pussegoda, Sindew Mekasha Feleke, Dylan R Pillai

**Affiliations:** Departments of Pathology & Laboratory Medicine, Medicine, and Microbiology, Immunology and Infectious Diseases, University of Calgary, Calgary, Alberta, Canada; Departments of Pathology & Laboratory Medicine, Medicine, and Microbiology, Immunology and Infectious Diseases, University of Calgary, Calgary, Alberta, Canada; Departments of Pathology & Laboratory Medicine, Medicine, and Microbiology, Immunology and Infectious Diseases, University of Calgary, Calgary, Alberta, Canada; Departments of Pathology & Laboratory Medicine, Medicine, and Microbiology, Immunology and Infectious Diseases, University of Calgary, Calgary, Alberta, Canada; Department of Bacteriology, Parasitology, and Zoonoses, Ethiopia Public Health Institute, Addis Ababa, Ethiopia; Department of Bacteriology, Parasitology, and Zoonoses, Ethiopia Public Health Institute, Addis Ababa, Ethiopia; Departments of Pathology & Laboratory Medicine, Medicine, and Microbiology, Immunology and Infectious Diseases, University of Calgary, Calgary, Alberta, Canada; Undergraduate Studies, University of Western Australia, Perth, Australia; Department of Bacteriology, Parasitology, and Zoonoses, Ethiopia Public Health Institute, Addis Ababa, Ethiopia; Department of Environment & Genetics, La Trobe University, Melbourne, Victoria, Australia; Departments of Pathology & Laboratory Medicine, Medicine, and Microbiology, Immunology and Infectious Diseases, University of Calgary, Calgary, Alberta, Canada

**Keywords:** malaria, histidine-rich protein 2, rapid diagnostic tests, artemisinin, kelch13

## Abstract

Malaria elimination relies on detection of *Plasmodium falciparum* histidine-rich proteins 2/3 (HRP2/3) through rapid diagnostic tests (RDTs) and treatment with artemisinin combination therapies (ACTs). Data from the Horn of Africa suggest increasing *hrp2/3* gene deletions and ACT partial resistance *kelch13* (*k13*) mutations. To assess this, 233 samples collected during a national survey from 7 regions of Ethiopia were studied for *hrp2/3* deletions with droplet digital polymerase chain reaction (ddPCR) and *k13* mutations with DNA sequencing. Approximately 22% of the study population harbored complete *hrp2/3* deletions by ddPCR. Thirty-two of 44 of *k13* single-nucleotide polymorphisms identified were R622I associated with ACT partial resistance. Both *hrp2/3* deletions and *k13* mutations associated with ACT partial resistance appear to be co-occurring, especially in Northwest Ethiopia. Ongoing national surveillance relying on accurate laboratory methods are required to elaborate the genetic diversity of *P. falciparum*.

## Teaser

Malaria screen and treat often depend on rapid diagnostic testing and artemisinin combination therapy, which is under threat from parasite gene variants. We report countrywide data from Ethiopia detailing *hrp2/3* deletions and *kelch13* mutation prevalence, using highly precise genetic methods.

*Plasmodium falciparum* causes most human malaria cases and caused 94% of total malaria-related deaths in the World Health Organization (WHO) African Region in 2022 [1, 2]. Current malaria diagnostic approaches rely either on direct identification of the parasite in blood samples with light microscopy or on rapid diagnostic tests (RDTs) [3]. The ease of use and availability of RDTs through subsidies has led to their broad implementation. It is now estimated that ≥300 million RDTs are used in malaria control programs each year [1]. RDTs work by detecting the *P. falciparum* histidine-rich protein-2 (HRP2) protein (which is specific to *P. falciparum*) or by detecting pan-*Plasmodium* proteins (lactate dehydrogenase or aldolase, or their species-specific variant). Of these, HRP2-based RDTs are the most broadly deployed [4].

A report in 2010 from Peru revealed parasites with the *hrp2* gene deleted resulting in the escape from diagnosis with HRP2 RDTs [3]. Since then, *hrp2/3-*deleted parasites have also been reported in sub-Saharan Africa. Systematic reviews suggest an increase in prevalence of *hrp2/3* gene deletions between 2018 and 2022 (from 17% to 21.3%, respectively) [5]. In their 2021 report, the WHO emphasized that *hrp2/3* deletions are among the major threats to malaria control and elimination programs [1]. The gene *hrp2* is located on chromosome 8, with exon 1 being 69 base pairs long and exon 2, 848 base pairs long. The gene *hrp3* is located on chromosome 13, with exon 1 also being 69 base pairs long and exon 2, 758 base pairs long [6]. The gene *k13* is located on chromosome 13. The nested polymerase chain reaction (PCR) region of the sequenced *k13* propellor domain is 848 base pairs long [7].

Confirmed *P. falciparum* cases are mostly treated by artemisinin combination therapies (ACTs). Delayed clearance of artemisinin was first observed in 2008 in Western Cambodia [8]. Single-nucleotide polymorphisms (SNPs) in the *pfkelch13* gene (*k13)* coding for the K13 protein propellor domain of *P. falciparum* were identified as the key mutations for delayed parasite clearance following ACT [9]. In more recent years, reports have emerged that ACT treatment failure is occurring in Uganda and Rwanda [10, 11]. Slow clearance rates are associated with recrudescence, which ultimately reflects the loss of efficacy of ACTs. ACT therapeutic efficacy studies are required to continually assess the clinical utility of this treatment regimen. In addition, molecular epidemiology surveys for the prevalence of *k13* mutations are required.

Infections with *Plasmodium,* and particularly *P. falciparum,* are polyclonal; that is, multiple parasite clones are infecting the human host [12]. These polyclonal infections can harbor different *hrp2* genotypes (deleted/nondeleted or different portion of the *hrp2* gene deleted), which might lead to underestimation or overestimation of *hrp2* deletion prevalence with conventional screening approaches such as PCR, which only amplifies the most abundant clone. Consequently, a limitation of many *hrp2/3* deletion studies is the presence of multiple clones within a clinical sample. PCR-based methods can lead to either false-positives (*hrp2* presence observed, while the isolate might contain *hrp2-*deleted clones) or false-negatives (*hrp2* deletion observed when a sample contains wild-type parasites). Accurately capturing the prevalence of *hrp2/3* deletion within a population is paramount to public health policies, particularly as the WHO recommends that areas with >5% prevalence of *hrp2* deletion should implement non–HRP2-based RDTs for malaria diagnostics [1].

The co-occurrence of diagnostic and treatment resistant parasites is threatening malaria control and elimination programs. The mechanisms and evolutionary forces driving the spread of these mutants are poorly understood. Recent studies conducted in Ethiopia has further emphasized that parasites are evolving to “escape” HRP2-based diagnostics with deletion in the *hrp2/3* genes [13–18]. An even more concerning finding from Mihreteab et al [19], published in 2023, shows an increased prevalence of artemisinin-resistant *hrp2*-deleted parasites over the past 5 years in neighboring Eritrea. Our study provides further evidence that these two phenotypes “drug and diagnostic resistance” are occurring in the region. However, the exact genetic and evolutionary mechanism of this coexistence remains to be elucidated. To this end, in the present countrywide cross-sectional study, we explored the co-occurrence of *hrp2/3* deletions and *k13* mutations by using a subset of samples from the Ethiopian national malaria survey. We also implemented a quantitative assay based on droplet digital PCR (ddPCR) technology to accurately quantify the proportion of *hrp2/3-*deleted parasites within a sample.

## METHODS

### Clinical Sample Selection

This study stems from a national cross-sectional study cohort performed by the Ethiopia Public Health Institute (EPHI) across 7 of the 9 national regional states in Ethiopia, following the WHO protocol [20]. Additional samples were collected within the malaria peak season (August–December) between 2017 to 2018 for Amhara, Tigray, and Gambella and between 2020 to 2022 for Afar, Somali, Oromia, and the Southern Nations, Nationalities, and Peoples’ Region (SNNPR). Participants were included through passive enrollment of the population for those presenting symptoms of malaria, such as fever, headache, joint pain, feeling cold, nausea, and/or poor appetite.

Whole-blood samples from patients testing negative after screening with a CareStart Pf/Pv (HRP2/Pv-pDH) RDT (Access Bio; catalog no. RM VM-02571) but positive by SD Bioline Malaria Ag P.f.(HRP2/2/Pf-LDH) RDT (Alere; catalog no. 05FK90) were preserved as dried blood spots (DBSs) on Whatman 3MM paper (Cytiva), together with 20% concordant positive samples in 2017. In the 2021 survey, CareStart Pf/Pv (HRP2/Pv-pDH) RDT–negative and microscopy-positive samples were collected using DBSs together with 20% concordant samples. A subset of deidentified DBSs (representative of 7 regional states of Ethiopia) was sent to the University of Calgary for downstream genetic analysis. Ethical approval was obtained from the EPHI Institutional Review Board (protocol EPHI-IRB-033-2017) and WHO Research Ethics Review Committee (protocol ERC.0003174 001). Genetic analysis of deidentified samples was conducted with ethical approval from the Conjoint Health Research Ethics Board at the University of Calgary (REB21-1059). Sample transfer was facilitated through a Memorandum of Understanding and Materials Transfer Agreement between University of Calgary and EPHI.

### DNA Extraction and Preservation

DNA was extracted from preserved DBSs. All extractions were performed by systematically hole-punching the DBS and using QIAamp DNA Blood Kits (Qiagen), according to the manufacturer’s recommendations. The obtained DNA was eluted in 50 µL of nuclease-free water (VWR) and preserved at −80°C until further use.

### Genomic DNA Controls for Molecular Assays

The following genomic DNA controls were used in each ddPCR and end-point PCR assay: MRA-102 (3D7, wild type), MRA-156 (DD2, Δ*hrp2*), and MRA-155 (Hb3, Δ*hrp3*) diluted at 100 genome copies/µL), alongside a mix of MRA-102 and MRA-156 for the *hrp2* and MRA-102 and MRA-155 for the *hrp3* assay. For the ddPCR assay optimization, mixtures of MRA-102, MRA-156, and MRA-155 were analyzed at concentrations of 1000, 500, 100, and 10 genomic DNA copies**/**µL at different relative ratios (90:10, 75:25, 50:50, 75:25, and 10:90, respectively). Mixtures at each concentration were run in triplicate, and the ratio of *hrp2/3* to serine-transfer RNA (tRNA) ligase was analyzed with their theoretical values to ensure the methods robustness. The following reagents were obtained through BEI Resources: *P. falciparum*, strain 3D7, MRA-102 (contributed by Daniel J. Carucci) and strain Dd2, MRA-156G, HB3, and MRA-155G (contributed by Thomas E. Wellems).

### Detection of *hrp2/3* Deletions With PCR and ddPCR

End-point *hrp2/3* PCR analysis was performed on the selected samples as described elsewhere [13, 21]. The *hrp2/3* deletion assays were performed using the ddPCR system from Bio-Rad (Bio-Rad), by modification of the ddPCR assay described elsewhere [22]. Modifications include that only droplet counts >10 000 per reaction were retained for downstream analysis. Ratios of *hrp2/3* to *tRNA ligase* that were >0.75 were said to have no deletion present. Ratios between 0.75 and 0.25 were labeled as mixed infections, and ratios <0.25 were said to have a full deletion present. Samples were included if ≥5 *tRNA ligase* droplets were present to show that sufficient parasitic DNA material was present for analysis. Of the 455 selected samples, 322 met the ≥5 *tRNA ligase* droplet threshold requirement for ddPCR analysis and were also evaluated with end-point PCR by screening for the exon 2 for both *hrp2* and *hrp3* genes. Of the 322 samples analyzed using ddPCR and PCR for *hrp2* and *hrp3*, 47 lacked sufficient genomic DNA for further analysis. A total of 275 samples underwent *k13* nested PCR and Sanger sequencing, resulting in successful sequencing of 233 samples. However, *k13* sequencing failed in 42 samples. Supplementary Tables 1, and 2 show the primers sequences used for PCR and ddPCR in the current study.

### *k13* Sequencing and SNPs

The *k13* propeller domain was amplified by nested PCR, as described elsewhere [9, 23]. PCR products were run on 2% agarose gel with a 1-kb ladder (New England Biolabs), and the bands at 849 base pairs were purified using the QI Quick Gel Extraction Kit (Qiagen). Bidirectional Sanger sequencing of the purified PCR products was performed using BigDye Terminator chemistry with an Applied Biosystems 3730XL instrument at the Centre for Health Genomics and Informatics (University of Calgary). Sequencing results were assessed individually by analyzing both strands with an MAFFT local pairwise alignment tool on a Benchling cloud-based platform (MAFFT version 7) against the *P. falciparum* 3D7 *k13* reference sequence (PlasmoDB: PF3D7_1343700). Supplementary Table 3 shows the primer sequences used for nested PCR.

### Principal Component Analysis of *hrp2/3* and *k13* Status

Across all samples sequenced for *k13*, a local alignment was performed using MAFFT, version 7, with default parameters. A dissimilarity matrix was constructed, and the principal components were depicted to reduce multidimensionality across samples.

### Statistical Analysis

Differences were considered statistically significant at *P* < .05. All statistical analyses were carried out using R (version 4.2.2.), Rstudio (version 1.3.1056) and GraphPad Prism (version 9.4.1) software. For distributions with continuous variables a Shapiro-Wilk test and an unpaired 2-tailed *t* test or Mann-Whitney test was performed to test for normality. For categorical variables, a 2 × 2 contingency table was generated, followed by a 2-tailed Fisher exact test. A Kruskal-Wallis test with repeated measures was performed when >2 distributions were present. A permutational multivariate analysis of variance with 999 permutations was carried out across the principal component analysis (PCA) to determine whether significance between centroids exist.

## RESULTS

### Samples and Study Population

**Figure 1.**
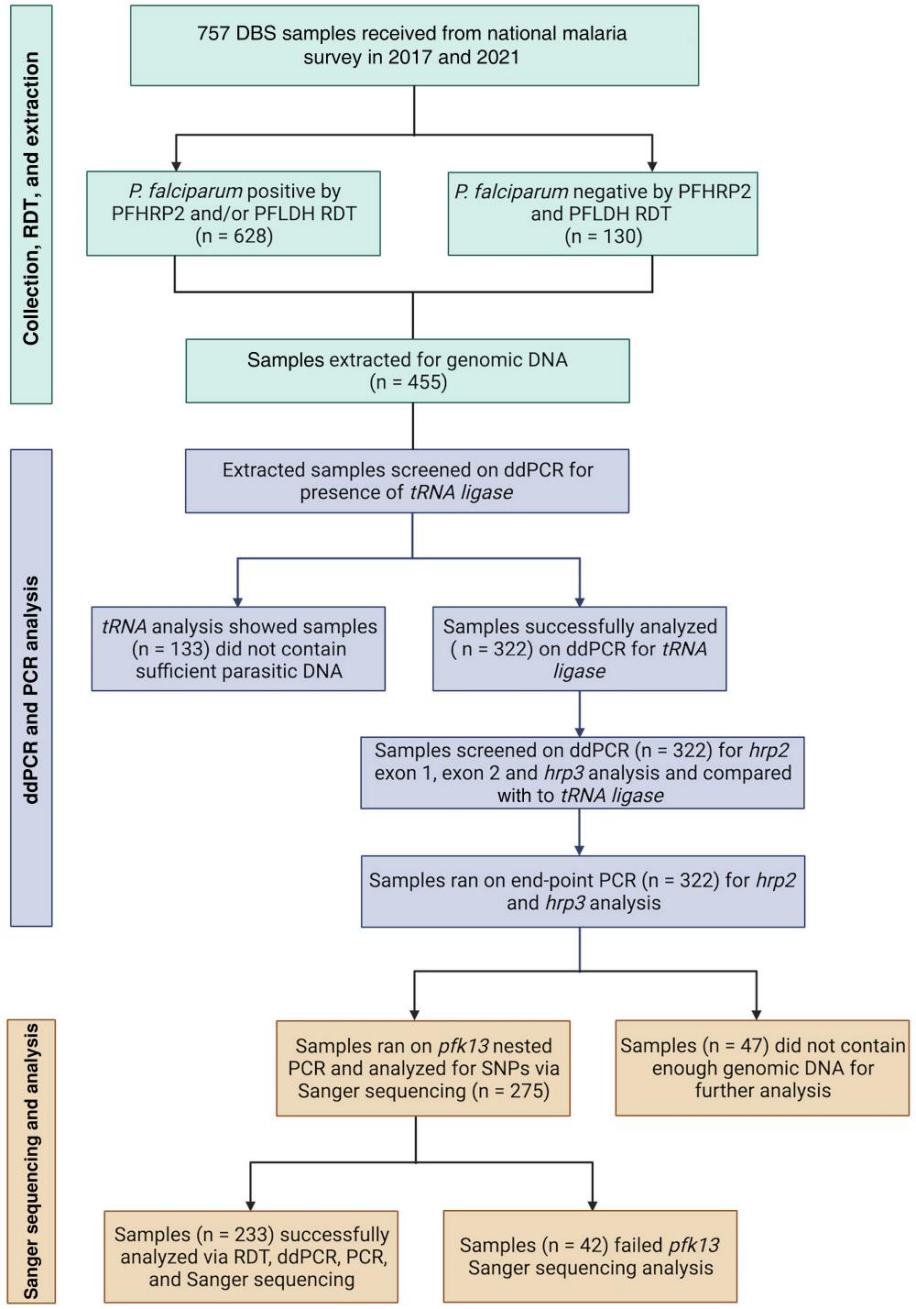
Study workflow and sample selection. A total of 757 dried blood spot (DBS) samples were received from the malaria surveys form 2017 and 2021 across Afar, Gambella, Oromia, the Southern Nations, Nationalities, and Peoples’ Region (SNNPR), and Somali regions. A total of 233 samples were analyzed with the 3 molecular tools implemented in this study: *hrp2/3* end-point polymerase chain reaction (PCR), droplet digital PCR (ddPCR) quantification of *hrp2* and *hrp3*, and *k13* sequencing. Abbreviations: *P. falciparum*, *Plasmodium falciparum*; RDT, rapid diagnostic test.

A total of 455 of 757 samples were selected for downstream analysis after statistical evaluation to ensure no geographic bias introduced (data not shown) (see Figure 1). The population selected for analysis had a sex ratio of 70.63% male to 29.37% female patients, an age range of 12–25.75 years, and a median parasite density of 2144**/**µL (range, 379.1–8419.9**/**µL) (see Table 1). These 455 samples were analyzed with the 3 molecular tools implemented in this study (*hrp2/3* end-point PCR, ddPCR quantification of *hrp2* and *hrp3*, and *k13* sequencing).

### Mixed Genotypes Revealed by PCR Versus ddPCR Analysis

Of the 233 samples analyzed, *hrp2/3* PCR results showed that 162 samples (69.53%), 71 (30.47%) were negative for *hrp2,* and 169 (72.53%) were negative for *hrp3.* 54 samples (23.18%) were positive for both *hrp2* and *hrp3,* and 61 (26.18%) were negative for both *hrp2* and *hrp3*. When the samples were analyzed with ddPCR, 55 (23.61%) showed full *hrp2/3* deletions (meaning that all the clones detected in these samples were deleted for the targeted gene) in *hrp2* exon 1, 51 (21.89%) in *hrp2* exon 2, and 161 (69.10%) in *hrp3*. Furthermore, the fine resolution of ddPCR allowed for the identification of samples containing mixed genotypes, meaning that multiples clones within the samples present a differing *hrp2* deletion status.

**Figure 2.**
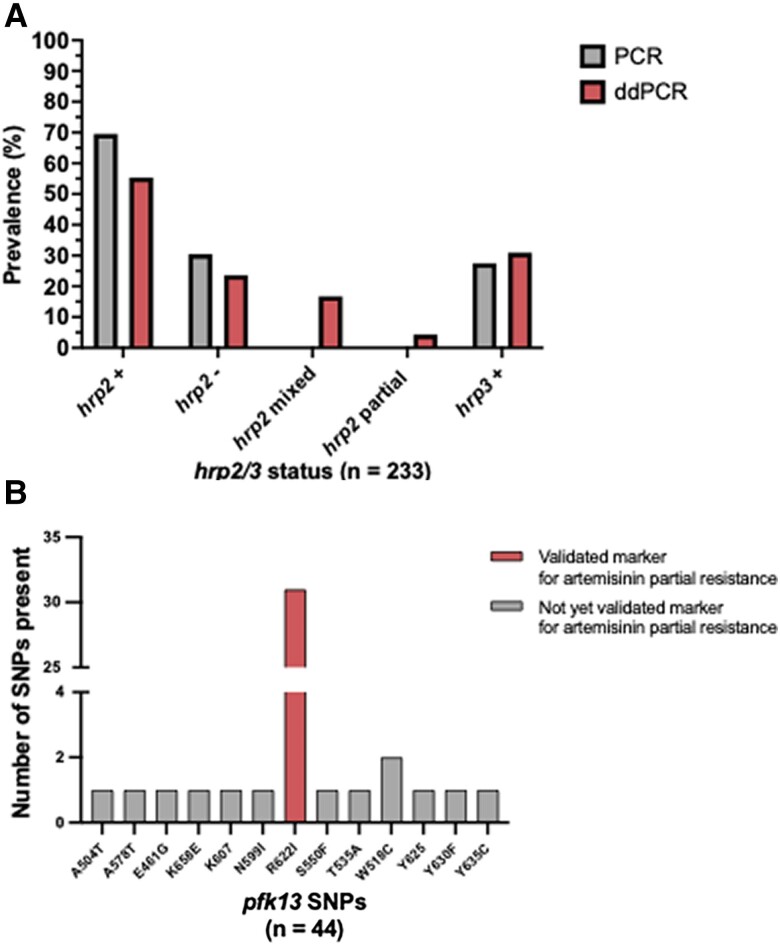
Prevalence of *hrp2/3* deletions in relation to polymerase chain reaction (PCR) and droplet digital PCR (ddPCR) and *k13* single-nucleotide polymorphism (SNP) sequencing results. *A*, Differences in *hrp2/3* status between PCR and ddPCR. PCR estimated that 30.47% (71 of 233) samples contained *hrp2* deletions, compared with 23.61% (55 of 233) for ddPCR. While PCR can only distinguish *hrp2* status only by full deletion (*hrp2*^−^) or no deletion (*hrp2*^+^), ddPCR can distinguish *hrp2* by no deletion, full deletion, mixed (16.74%), or partial (4.29%). *B*, Among the 233 samples analyzed, a total of 13 unique *k13* SNPs were identified across 44 samples (all nonsynonymous). The most prevalent *k13* SNP detected was R622I 70.45% [31 of 44], Only 1 other SNP, W518C, was detected in ≥1 sample 4.54% [2 of 44].

Mixed genotypes represented 16.74% of samples (39 of 233) for *hrp2.* In addition, 4.29% of samples (10 of 233) had partial deletions in *hrp2* (7 in exon 1 and 3 in exon 2). Only 7.73% of samples (18 of 233) had mixed *hrp3* infections. As the *hrp3* status was deemed less significant from a RDT reactivity perspective, downstream analysis grouped *hrp3* as not deleted or deleted (with mixed infections listed as not deleted) (Figure 2*A*). Details on significant differences among the techniques used can be seen in the Supplementary materials (Supplementary Table 4). A total of 64 samples were negative for HRP2 by means of RDT, with ddPCR suggesting only 33 were truly negative for the exon encoding for the HRP2 protein in the RDT. Furthermore, PCR suggested that 36 samples were *hrp2* negative.

**Table 1.** Demographics and Selected Clinical Characteristics of the Study Population

Variable	Patients, No. (%)^a^ (n = 233)
Patient distribution	
Afar	7 (3.00)
Amhara	163 (69.95)
Gambella	5 (2.14)
Oromia	24 (10.30)
Somali	7 (3.00)
SNNPR	10 (4.29)
Tigray	36 (15.45)
Patient data	
Age, median (IQR), y	19 (12–25.75)
Sex	
Male	178 (70.63)
Female	74 (29.37)
Residence^b^	
Urban	63 (27.03)
Rural	162 (69.52)
Parasite density, median (IQR), parasites/µL	2144 (379.1–8419.9)
Symptoms	
Fever (>38.5°C)	225 (96.56)
Headache	223 (95.70)
Nausea^c^	136 (58.36)
Joint pain	203 (87.12)
Chills	155 (66.52)
Treatment^d^	
Any	49 (21.03)
ACT	36 (15.45)
Other^e^	13 (5.57)

Abbreviations: ACT, artemisinin combination therapy; IQR, interquartile range; SNNPR, Southern Nations, Nationalities, and Peoples’ Region.

^a^Data represent no. (%) of patients unless otherwise specified.

^b^Residential data were missing for 8 persons.

^c^Information on nausea was missing for 1 person.

^d^Treatment was recorded as “present” if the patient received an antimalarial in the 2 weeks before study enrollment. No history of treatment was recorded for 1 patient; no history of treatment was recorded for 2 persons.

^e^Other types of antimalarials include chloroquine, artemether-lumefantrine, and quinine.

### Detection of SNPs in the *kelch13* Propeller Domains

**Figure 3.**
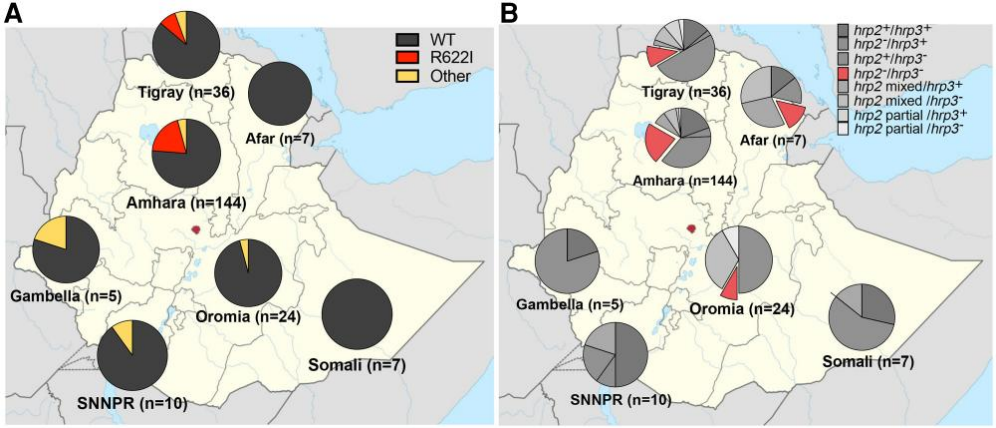
Regional distribution of *hrp2/3* and *k13* genotypes. Plots demonstrate distribution of samples by region in Ethiopia and their associated *k13* and *hrp2/3* status. *A*, Presence of *k13* single-nucleotide polymorphisms in the propellor domain, with red highlighting the presence of the R622I mutation. Abbreviations: SNNPR, Southern Nations, Nationalities, and Peoples’ Region; WT, wild type. *B*, *hrp2/3* status of each region obtained with digital droplet polymerase chain reaction, with red highlighting samples that contain both *hrp2* and *hrp3* deletions (*hrp2*^−^ and *hrp3*^−^), representing complete loss of *hrp2/3*.

A total of 13 unique nonsynonymous SNPs were identified among 44 total samples containing SNPs (Figure 2*B*). Of these identified SNPs in the K13 propellor domain, 31 samples encoded for R622I, a validated marker for artemisinin partial resistance. W518C was the only other SNP to be found more than once (n = 2). The region containing the highest proportion of the R662I mutations was found in Amhara (19.44% [28 of 144]). The Tigray region was the only other region where the R622I mutation was found (8.33% [3 of 36]). All regions other than Afar and Somali contained ≥1 SNP. Two samples contained 2 different SNPs, one in Amhara (R622I and Y625) and the other in Gambella (Y630F, A504T). Among the samples presenting the R622I SNP, 12.9% (4 of 31) were deleted for *hrp2* exon 1 and 9.7% (3 of 31) *hrp2* exon 2 deletion, and 2 samples (6.45%) had deletions in both *hrp2* exons. In addition, 90.3% of samples (28 of 31) containing an R622I SNP also had an *hrp3* deletion (Figure 3*A* and *B*).

### PCA of *hrp2/3* and *k13* Status

**Figure 4.**
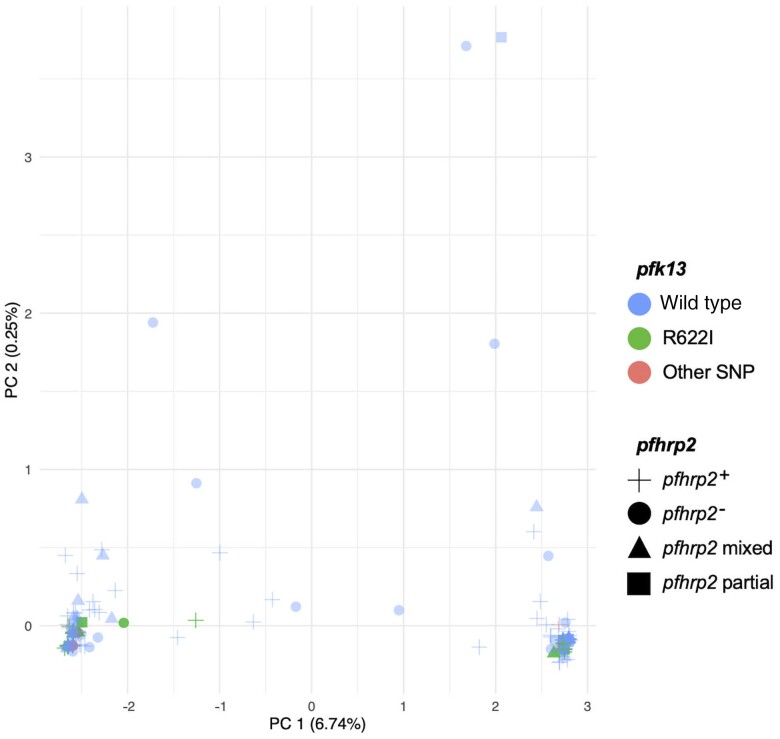
Principal component analysis (PCA) of *pfhrp2/3* and *pfk13* status. The constructed dissimilarity matrix and the principal components (PCs) in the graph display the 233 samples analyzed for pf*k13* single-nucleotide polymorphisms (SNP) (wild type in blue, R622I in green, and other SNPs in red). The *hrp2* status assessed by droplet digital polymerase chain reaction is represented by shapes; green circles represent samples that were negative for *pfhrp2* and encoded an R622I SNP in the *p*f*k13* propellor domain. PCA explains that the sequence dissimilarity of the *pfk13* propellor domain does not differ significantly by *pfhrp2* status or R622I.

PCA demonstrated that the sequence dissimilarity of the *k13* propellor domain did not differ significantly by *hrp2* status or R622I (Figure 4). In support of this, in separate analysis, no significant association was found between the presence of *hrp2* assessed by ddPCR, regardless of the extent of the deletion (positive, mixed, or partial, negative; n = 168), and the presence of any R622I SNPs in the *k13* propeller among RDT-positive samples (*P* = .15). The *hrp2*-positive/partial/mixed samples had a higher rate of encoding an R622I SNP compared with exon 1 and 2 *hrp2*-negative samples (*P* = .03). No significant association was observed between *hrp3* deletions (n = 215) and the presence of SNP/R622I in the *k13* propeller domain (*P* = .09).

## DISCUSSION

In the current study using PCR and ddPCR methodologies, *hrp2/3* PCR assays suggest that 30.47% of the samples analyzed are HRP2-deleted parasites, while this number decreases to 23.61% for *hrp2* exon 1 and 21.89% for exon 2 when analyzed with ddPCR. The reason for this difference in deletion estimation (6.86%–8.58%) may be attributed to the facts that 16.74% of samples retained polyclonal infections and 4.29% elaborated partial deletions. Furthermore, since most *hrp2* PCR assays exclude the detection of exon 1 [20], the combination of polyclonal infections, partial and full deletions, and lower sensitivity can cause an overestimation of *hrp2* deletions. These differences can also be attributed to the higher analytical sensitivity of ddPCR.

We also analyzed *hrp3* deletions with ddPCR and determined that almost 69.10% of samples contained HRP3-deleted parasites. A trend in high amounts of HRP3-deleted parasites in the horn of Africa has been reported before by *hrp3* PCR [13, 24]. ddPCR analysis also showed that 17.16% of the samples were *hrp2/3* double-deleted parasites. However, of these 17.16% double-deleted parasites, 67.50% were negative for HRP2 via RDT. This shows an increase compared with previous studies [15–17, 25]. Taken together, our findings and those of others suggest that deletion rates exceed the 5% WHO recommendation for HRP2-deleted infections in certain regions of Ethiopia [13].

As suggested by recent studies in Ethiopia and its neighboring country, Eritrea [14, 19], the presence of partial artemisinin resistance in Ethiopia is increasing due to occurrence of SNPs in the *k13* gene. A worrying trend is the increase in the prevalence of the R622I *k13* mutation, first reported in Ethiopia in 2015 [26]. In 2022, the WHO classified R622I as a validated marker for partial resistance to artemisinin [1, 27]. Other than R622I, no other SNP detected in this study has been validated by the WHO for artemisinin resistance. Only one other SNP identified in this study, W518C (previously reported in Kenya) [28], was identified more than once (2 of 44 [4.54%]). All but 1 SNP, Y625 (2.27% [1 of 44]), were previously reported in earlier studies, with 2 being previously reported in Ethiopia, N599I (2.27% [1 of 44]) and S550F (2.27% [1 of 44]). All but 1 SNP, T535A (2.27% [1 of 44]) had been previously reported in Africa.

As reported by Fola *et al* [14] and Mihreteab *et al* [19], the interplay of *hrp2/3* deletions and *k13* SNPs co-occurring to resist both detection against HRP2 RDTs and ACT treatment may be increasing due to selective pressure. This was noted in our study, in which 10.26% of double-deleted *hrp2/3* samples contained a *k13* SNP, with 5.13% being R622I. Moreover, 13.73% of samples that contained an exon 2 deletion of *hrp2*, the gene that expresses the antigen used for detection in HRP2 RDTs, also encoded a *k13* SNP, with 5.88% encoding R622I. Interestingly, it has been reported that *hrp2/3* deletions may be associated with drug resistance [29, 30], as HRP2/3 plays a role in the digestion/metabolism of hemoglobin to hemozoin in *P. falciparum*. By deleting HRP2/3, the metabolism of heme is slowed [29, 30].

Notably, hemoglobin by-products activate artemisinin, so by slowing hemoglobin digestion, the susceptibility of the parasite to ACT is reduced [31–33]. However, the associated fitness cost of these deletions might slow the rate of spread of these mutant genotypes [34, 35]. In addition, the role of the *k13* gene in drug resistance has also been associated with hemoglobin digestion [31, 36]. Thus, both *hrp2/3* deletions and *k13* mutations may arguably have similar roles in providing *P. falciparum* resistance to ACT. Nevertheless, our findings indicate that these deletion and mutations are co-occurring without a clear statistical association. Further studies that incorporate population genetic signatures are warranted to confirm the exact evolutionary relationship between the 2 phenotypes.

There are some limitations to our study. Our data set was predominantly samples from the Amhara region, which is the largest population, but analysis of more samples from the 6 other regions in the future could provide a better national assessment of *hrp2/3* and *k13* status. Furthermore, the inclusion of next-generation sequencing of *k13* SNPs, such as amplicon deep sequencing, may further elucidate the genetic complexity of ACT resistance and HRP2 absence, as recently demonstrated by studies on atovaquone-proguanil resistance [37].

In conclusion, data presented here demonstrate that ddPCR provides a better estimate of the true prevalence of *hrp2/3* deletions and of how factors such as partial deletions and mixed infections may affect determination of the percentage of parasites that do not encode *hrp2/3*. We have also highlighted that the *k13* propellor domain SNP that provides partial resistance to artemisinin, R622I, is becoming more prevalent in certain regions of Ethiopia. Most importantly, we have indicated that co-occurrence of *hrp2/3* deletions with *k13* SNPs may be more complex than initially described. Factors such as polyclonal infections and the subsequent difficulties in identifying *hrp2/3* deletions may affect the prevalence of co-occurrence between these genes. Further research is warranted to establish the true relationship between *hrp2/3* deletions and *k13* mutations.

## Supplementary Data

Supplementary materials are available at *The Journal of Infectious Diseases* online (http://jid.oxfordjournals.org/). Supplementary materials consist of data provided by the author that are published to benefit the reader. The posted materials are not copyedited. The contents of all supplementary data are the sole responsibility of the authors. Questions or messages regarding errors should be addressed to the author.

## Supplementary Material

jiae373_Supplementary_Data

## Notes

*Acknowledgments.* We thank the EPHI surveyor staff for the collection of samples. We also thank Lisa Oberding for help with developing the radial multiplex ddPCR assay and Rebecca Walchyshyn from the Centre of Health Genomics and Informatics (University of Calgary) for help with the sequencing of our samples. We thank MSF Holland for supporting sample collection in the Gambella region.

***Author contributions*.** A.A., B.M.T., and S.M.F collected clinical data, coordinated the national survey, and performed routine laboratory analysis. C. K., J. B. G., and A. W. managed the samples. C. K. and J. B. G. adapted the ddPCR assay. C. K., J. B. G., A. W., and E. P. performed the ddPCR assay. J. B. G., S. A., and A. W. performed the PCR analysis. J. B. G. and S. A. performed the *k13* sequencing laboratory work, and J. B. G. and D. C. M. conducted bioinformatic analysis. C. K., J. B. G., and S. A. prepared the initial draft. J. B. G. and D. C. M. performed the statistical analysis, and D. C. M. performed the PCA. S. M. F., and D. R. P. supervised, designed, and obtained funding for the different elements of this study. All authors critically reviewed the manuscript and agreed on the final form.

***Financial support*.** This work was supported by the University of Calgary (VPR Grant, D.R.P), Grand Challenges Canada (TTS Grant, D.R.P), and Ethiopian Public Health Institute (Grant EPHI5405, S.M.F).
