# Supplementary material for: A Countrywide Survey of hrp2/3 Deletions and kelch13 Mutations Co-occurrence in Ethiopia
Source: J Infect Dis. 2024 Jul 31;230(6):e1394–401. doi: 10.1093/infdis/jiae373 (PMC11646589; doi:10.1093/infdis/jiae373)
Supplement: jiae373_Supplementary_Data [file jiae373_supplementary_data.docx]

**Supplementary Tables:**

### Supplementary Table 1. Primer Supplementary Table for PCR *hrp2/3* assays

| **PCR - *hrp2* exon 2 & *hrp3*** | | | |
| --- | --- | --- | --- |
| **Primer** | **Sequence** | **concentration** | **adapted from** |
| *hrp2* forward primer | CAAAAGGACTTAATTTAAATAAGAG | 200 nM | Baker J et al. J infect Dis 2005. 192:870-877 [1] |
| *hrp2* reverse primer | AATAAATTTAATGGCGTAGGCA | 200 nM | Baker J et al. J infect Dis 2005. 192:870-877 [1] |
| *hrp3* forward primer | AATGCAAAAGGACTTAATTC | 200 nM | Baker J et al. J infect Dis 2005. 192:870-877 [1] |
| *hrp3* reverse primer | TGGTGTAAGTGATGCGTAGT | 200 nM | Baker J et al. J infect Dis 2005. 192:870-877 [1] |

### Supplementary Table 2. Primer Supplementary Table for ddPCR *hrp2/3* assays

| **ddPCR - *hrp2* exon 1 & 2, *hrp3,* and *tRNA ligase*** | | | |
| --- | --- | --- | --- |
| **Primer / Probe** | **sequence** | **concentration** | **adapted from** |
| *hrp2* exon 1 forward primer | ATATTTATACATTTTTGTTATTATTTCTTTTTC | 900 nM | Vera-Arias CA et al. Elife 2022. 10.7554 72083 [2] |
| *hrp2* exon 1 reverse primer | CGTTATCTAACAAAAGTACGGAG | 900 nM | Vera-Arias CA et al. Elife 2022. 10.7554 72084 [2] |
| *hrp2* exon 1 probe 1 | [FAM]CAAAAACGGCAGCGGATAATACTT[BHQ1] | 125 nM | Vera-Arias CA et al. Elife 2022. 10.7554 72085 [2] |
| *hrp2* exon 1 probe 2 | [HEX]CAAAAACGGCAGCGGATAATACTT[BHQ1] | 125 nM | Vera-Arias CA et al. Elife 2022. 10.7554 72086 [2] |
| *hrp2* exon 2 forward primer | CATTTTTAAATGCTTTTTTATTTTTATATAG | 900 nM | Vera-Arias CA et al. Elife 2022. 10.7554 72087 [2] |
| *hrp2* exon 2 reverse primer | CTTGAGTTTCGTGTAATAATCTC | 900 nM | Vera-Arias CA et al. Elife 2022. 10.7554 72088 [2] |
| *hrp2* exon 2 probe | [FAM]CGCATTTAATAATAACTTGTGTAGCAAAAATGC[BHQ1] | 250 nM | Vera-Arias CA et al. Elife 2022. 10.7554 72089 [2] |
| *hrp3* forward primer | ATGCTAATCACGGATTTCATTTTA | 900 nM | Vera-Arias CA et al. Elife 2022. 10.7554 72096 [2] |
| *hrp3* reverse primer | ATCGTCATGGTGAGAATCATC | 900 nM | Vera-Arias CA et al. Elife 2022. 10.7554 72097 [2] |
| *hrp3* probe | [FAM]CCTTCACGATAACAATTCCCATACTTTAC[BHGQ1] | 250 nM | Vera-Arias CA et al. Elife 2022. 10.7554 72098 |
| *tRNA* forward primer | CATCAAATGAAGATTTAACAAGAG | 900 nM | Vera-Arias CA et al. Elife 2022. 10.7554 72090 [2] |
| tRNA reverse primer | CTTTTTGATTCTATAGTTTCATCTTTATG | 900 nM | Vera-Arias CA et al. Elife 2022. 10.7554 72091[2] |
| tRNA probe | [HEX]CTACCTCAGAACAACCATTATGTGCT[BHQ1] | 250 nM | Vera-Arias CA et al. Elife 2022. 10.7554 72092 [2] |

### Supplementary Table 3. *k13* primary and nested PCR primers

| **PCR - *k13* Primary and Nested** | | | |
| --- | --- | --- | --- |
| **primer** | **Sequence** | **Concentration** | **adapted from** |
| *k13* Primary Forward | GGGAATCTGGTGGTAACAGC | 1 uM | Ariey F et al. Nature 2014. 10.1038 12876 [3] |
| *k13* Primary Reverse | CGGAGTGACCAAATCTGGGA | 1 uM | Ariey F et al. Nature 2014. 10.1038 12877 [3] |
| *k13* Nested Forward | GCCTTGTTGAAAGAAGCAGA | 1 uM | Ariey F et al. Nature 2014. 10.1038 12878 [3] |
| *k13* Nested Reverse | GCCAAGCTGCCATTCATTTG | 1 uM | Ariey F et al. Nature 2014. 10.1038 12879 [3] |

| Assays Compared | Both positive | Both negative | First positive, second negative | First negative, second positive | % of agreement | Cohen's K value | Cohen's K interpretation |
| --- | --- | --- | --- | --- | --- | --- | --- |
| ddPCR vs PCR | 145 | 35 | 36 | 17 | 77.25 | 0.42 | Moderate agreement |
| ddPCR vs RDT | 151 | 33 | 30 | 19 | 78.97 | 0.44 | Moderate agreement |
| RDT vs PCR | 135 | 36 | 35 | 29 | 72.77 | 0.34 | Fair agreement |
| ddPCR + RDT vs PCR | 123 | 28 | 28 | 5 | 82.07 | 0.52 | Moderate agreement |
| ddPCR + PCR vs RDT | 123 | 28 | 22 | 7 | 83.89 | 0.56 | Moderate agreement |
| RDT + PCR vs ddPCR | 123 | 28 | 12 | 7 | 88.82 | 0.68 | Substantial agreement |

### Supplementary Table 4. *Cohen’s Kappa calculation to calculate discrepencies among different methods*

### 0.01 – 0.20 = slight agreement

### 0.21 – 0.40 = fair agreement

### 0.41 – 0.60 = moderate agreement

### 0.61 – 0.80 = substantial agreement

### 0.81 – 1.00 = almost perfect or perfect agreement

### References

1. Baker J, McCarthy J, Gatton M, et al. Genetic diversity of Plasmodium falciparum histidine-rich protein 2 (PfHRP2) and its effect on the performance of PfHRP2-based rapid diagnostic tests. The Journal of infectious diseases **2005**; 192(5): 870-7.

2. Vera-Arias CA, Holzschuh A, Oduma CO, et al. High-throughput Plasmodium falciparum hrp2 and hrp3 gene deletion typing by digital PCR to monitor malaria rapid diagnostic test efficacy. Elife **2022**; 11: e72083.

3. Ariey F, Witkowski B, Amaratunga C, et al. A molecular marker of artemisinin-resistant Plasmodium falciparum malaria. Nature **2014**; 505(7481): 50-5.
